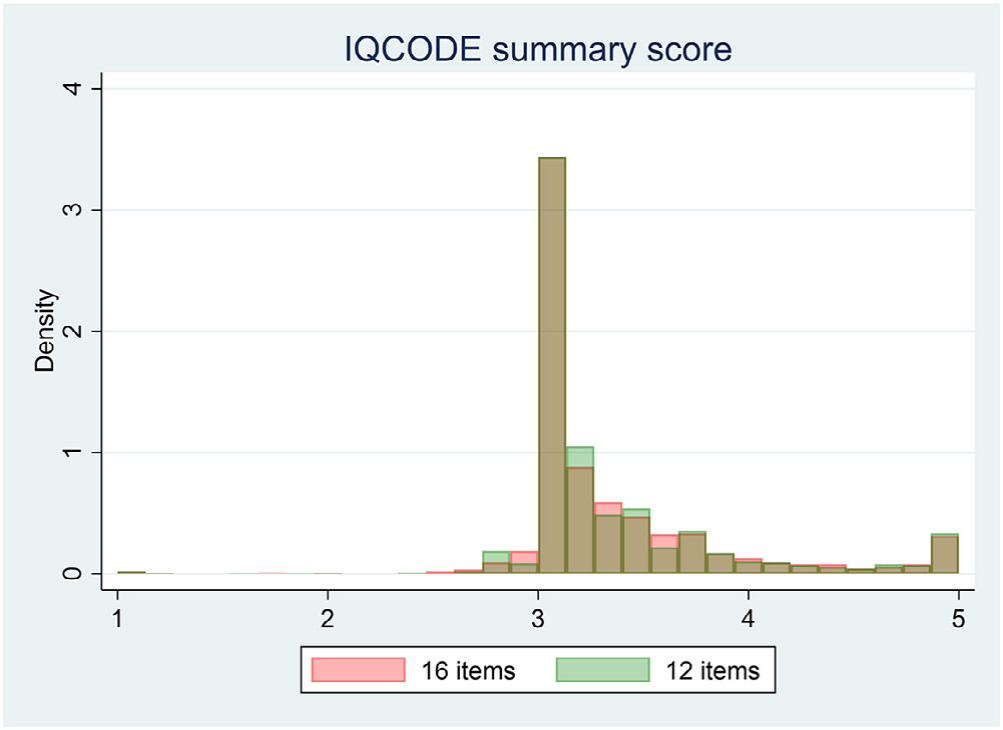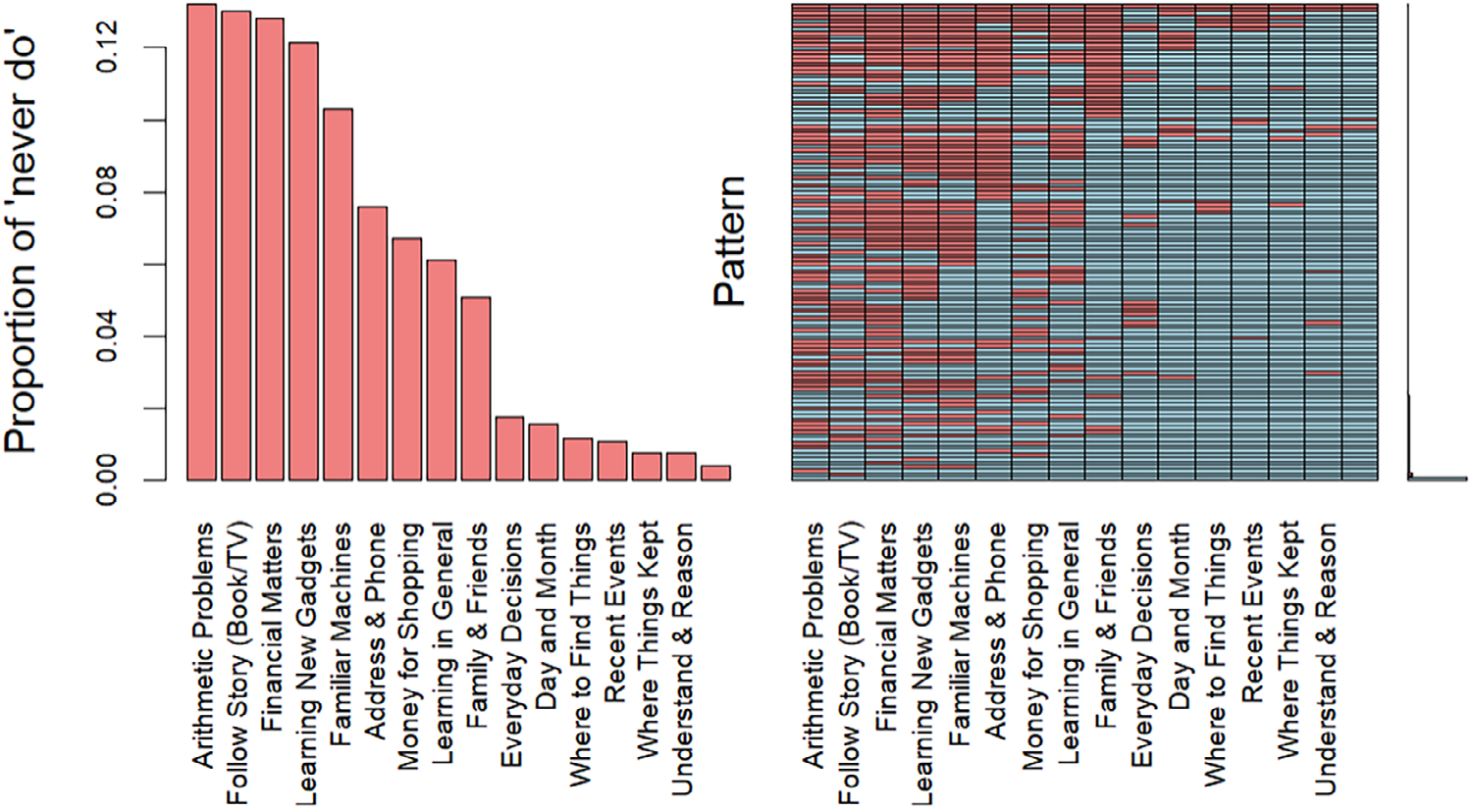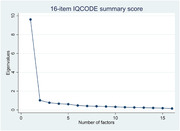# Performance of the Informant Questionnaire on Cognitive Decline for the Elderly (IQCODE) in Rural South Africa

**DOI:** 10.1002/alz.085510

**Published:** 2025-01-09

**Authors:** Muqi Guo, Tamara P. Taporoski, Meagan T. Farrell, Nomsa Mahlalehla, Lisa F. Berkman, Darina T. Bassil

**Affiliations:** ^1^ Harvard T.H. Chan School of Public Health, Cambridge, MA USA; ^2^ University of the Witwatersrand, Johannesburg South Africa

## Abstract

**Background:**

Informant reports are commonly regarded as reliable and supplemental alongside respondent cognitive assessments, particularly in low‐literacy settings with absent normative data. We evaluate the performance of the Informant Questionnaire on Cognitive Decline in the Elderly (IQCODE) in rural South Africa.

**Method:**

This study utilizes data from the Cognition and Dementia in a Longitudinal Health and Aging Study in South Africa (HAALSI‐HCAP). The study population includes 1,029 unique informants of 676 paired participants across two survey waves. Among them, 475 participants followed in both waves and 124 of them had the same informants. A Shangaan‐translated 16‐item IQCODE was surveyed among informants. We examined IQCODE completeness and scores, tested the single latent concept assumption of IQCODE when applied to South Africa using factory analysis, and assessed IQCODE inter‐item reliability and convergent validity. We finally examined whether using a different informant affects IQCODE score using a linear regression. Analyses were replicated after excluding items with higher proportion of reporting ‘participant never do’.

**Result:**

On average, informants aged 43.6 years and knew the paired participants for 32 years. Most informants were female (65%), participants’ children (48%), with above secondary degrees (45%), and lived with participants (66%). Informants averagely reported 0.9 items “participant never do”. Informants seeing participants daily versus those living together reported less “never do” items and give lower IQCODE score, controlling participants’ age, gender, and education. Children informants versus spouses tend to give higher IQCODE scores. Consistent with literature, IQCODE is a single concept with the first factor explaining 60% of total variance. IQCODE had excellent inter‐item reliability (Cronbach’s alpha = 0.94) and moderately correlated with participants’ recall memory scores. Using different informants does not affect IQCODE changes for the same participants over years. After excluding items with above 12% of informants reporting ‘participant never do’ (e.g., handling everyday arithmetic problems, financial matters, following a story, or learning new gadgets), results remained similar.

**Conclusion:**

Informant characteristics affects IQCODE scores but not completeness. Changing informants between waves does not impact IQCODE score. With excellent reliability and good validity even upon excluding several items, IQCODE is useful for assessing dementia among South African older adults.